# Oral health-related quality of life in adult patients with end-stage kidney diseases undergoing renal replacement therapy – a systematic review

**DOI:** 10.1186/s12882-020-01824-7

**Published:** 2020-04-29

**Authors:** Gerhard Schmalz, Susann Patschan, Daniel Patschan, Dirk Ziebolz

**Affiliations:** 1 Department of Cariology, Endodontology and Periodontology, University of Leipzig, Liebigstr. 12, D 04103 Leipzig, Germany; 2 Department of Cardiology, Angiology and Nephrology, Klinikum Brandenburg, Medizinische Hochschule Brandenburg, Neuruppin, Brandenburg Germany

**Keywords:** Oral health, Oral health-related quality of life, Renal replacement therapy, Haemodialysis, Kidney transplantation

## Abstract

**Background:**

The oral health of patients undergoing renal replacement therapy (RRT) is insufficient. Poor oral health and its components can affect the oral health-related quality of life (OHRQoL) of these patients. The aim of this systematic review was to assess the OHRQoL of adult patients under RRT.

**Methods:**

A systematic literature search was performed using the terms: dialysis OR “renal disease” OR kidney OR “renal failure” OR “kidney transplantation” OR hemodialysis OR “peritoneal dialysis” OR “renal replacement therapy” AND “oral health-related quality of life”, complemented by manual search. Clinical studies including adults (age ≥ 18 years) that were published between 2009 and 2019 were included in qualitative analysis.

**Results:**

Twelve out of 20 studies were included in the qualitative analysis. The majority (11/12 studies) included patients undergoing haemodialysis (HD), with a sample size between 47 and 512 participants. Two studies included patients after kidney transplantation. Only one-quarter of the investigations included a healthy control group. The overall OHRQoL was found to be reduced. The majority of studies found relationships between OHRQoL and different oral health parameters. Furthermore, several relationships between OHRQoL and general quality of life as well as disease related parameters including age, gender, diabetes, blood parameters and dialysis duration were found. OHRQoL subscales psychological/psychosocial impairment and pain were predominantly affected.

**Conclusions:**

Patients under RRT suffer from a reduced OHRQoL, which is potentially influenced by oral health and disease related parameters. Interdisciplinary dental care is needed and should consider both physical and psychosocial issues.

## Background

Renal disorders represent a global burden, whereby more than two million people in the world are treated for end-stage renal diseases [1]. These diseases often require renal replacement therapy (RRT), of which haemodialysis (HD) represents the most commonly used measure [2]. Recently, the incidence of RRT has decreased slightly, while patient survival has improved continuously [3]. The improvements in therapeutic measures during RRT and the decrease in mortality might lead to a change in the major challenge of care for these patients; thus, patient-related outcomes such as improving patients’ well-being and quality of life are of increasing importance [1, 3, 4]. It is known that health-related quality of life (HRQoL) is often reduced by RRT, especially for HD [4–6].

HRQoL is complex and affected by different factors, including oral conditions such as tooth loss, dental caries and periodontitis [7]. Therefore, a specific part of HRQoL with respect to the oral cavity is defined: the oral health-related quality of life (OHRQoL) [8]. This OHRQoL is a multidimensional model that provides information of the subjectively perceived influence of oral health conditions on patients’ oral function, pain, psychosocial impact and orofacial appearance [9]. OHRQoL is of clinical relevance for patients with RRT for two major reasons. On the one hand, patients with end-stage renal diseases, especially those receiving HD on a regular basis, suffer from an insufficient oral health status and oral behaviour [10]. Potential associations between renal insufficiency and HD with periodontal diseases have been reported [11]. Moreover, early tooth loss is observed, especially in patients undergoing HD [12]. Accordingly, a reduced OHRQoL caused by this increased prevalence of oral diseases is conceivable.

On the other hand, patients with end-stage renal disease suffer from a high emotional burden [13]. Patients undergoing HD are frequently affected by depressive disorders [14]. Related to the emotional burden, depression and anxiety, the overall HRQoL of the patients is reduced [4–6]. Accordingly, a negative effect of disease-related parameters and general emotional well-being on OHRQoL could be relevant. The extent to which the OHRQoL, as well as the general HRQoL, of patients with end-stage renal disease is influenced by oral health and general disease-related parameters is also clinically relevant. This information can help to improve the interdisciplinary dental care of patients with end-stage renal diseases undergoing RRT, as demanded in the literature [15].

This systematic review aimed to assess the OHRQoL of adult patients with end-stage renal diseases undergoing RRT (HD and KTx). The relationships between OHRQoL with oral health, HRQoL and disease-related parameters were evaluated. Furthermore, subscales of OHRQoL were considered. Based on this systematic review, implications for clinical management of the patients as well as recommendations for future research in the field are provided.

## Methods

A systematic search was performed in January 2020 based on the PubMed database using the following search terms: dialysis OR “renal disease” OR kidney OR “renal failure” OR “kidney transplantation” OR hemodialysis OR “peritoneal dialysis” OR “renal replacement therapy” AND “oral health-related quality of life”. The systematic search of these key terms was complemented by a manual literature search. All findings were screened and checked for their eligibility with regard to previously defined inclusion and exclusion criteria. Only full-text articles in the English language, published within the previous 10 years, i.e., between 1st January 2009 and 31st December 2019, were considered for inclusion in the analysis. Conditions for inclusion were the clinical examination of patients with end-stage renal diseases either treated with dialysis or kidney transplantation. Any form of OHRQoL measurement must have been applied and reported. Only studies with adult patients, i.e., participants with an age of at least 18 years, were included. For the qualitative analysis, the following major information was extracted from the included investigations:

- Type of treatment (dialysis or transplantation), year of publication, number of participants, study type, age, gender, disease duration
- Recruitment of a healthy control group for comparison of OHRQoL findings
- Examined oral health parameters and results, if applicable
- Type, results and validity of OHRQoL assessment
- Potential relationship between OHRQoL with general HRQoL, oral health and/or disease specific parameters
- Results for subscales of the OHRQoL measurements, if applicable

Clinical studies were screened for the defined inclusion and exclusion criteria. If studies included patients who were part of previously published investigations, they were checked to determine whether there were repetitious results. In this case, only the most recent examination was included and analysed. The systematic search was executed by two independent reviewers.

## Results

### Search findings

**Fig. 1:**
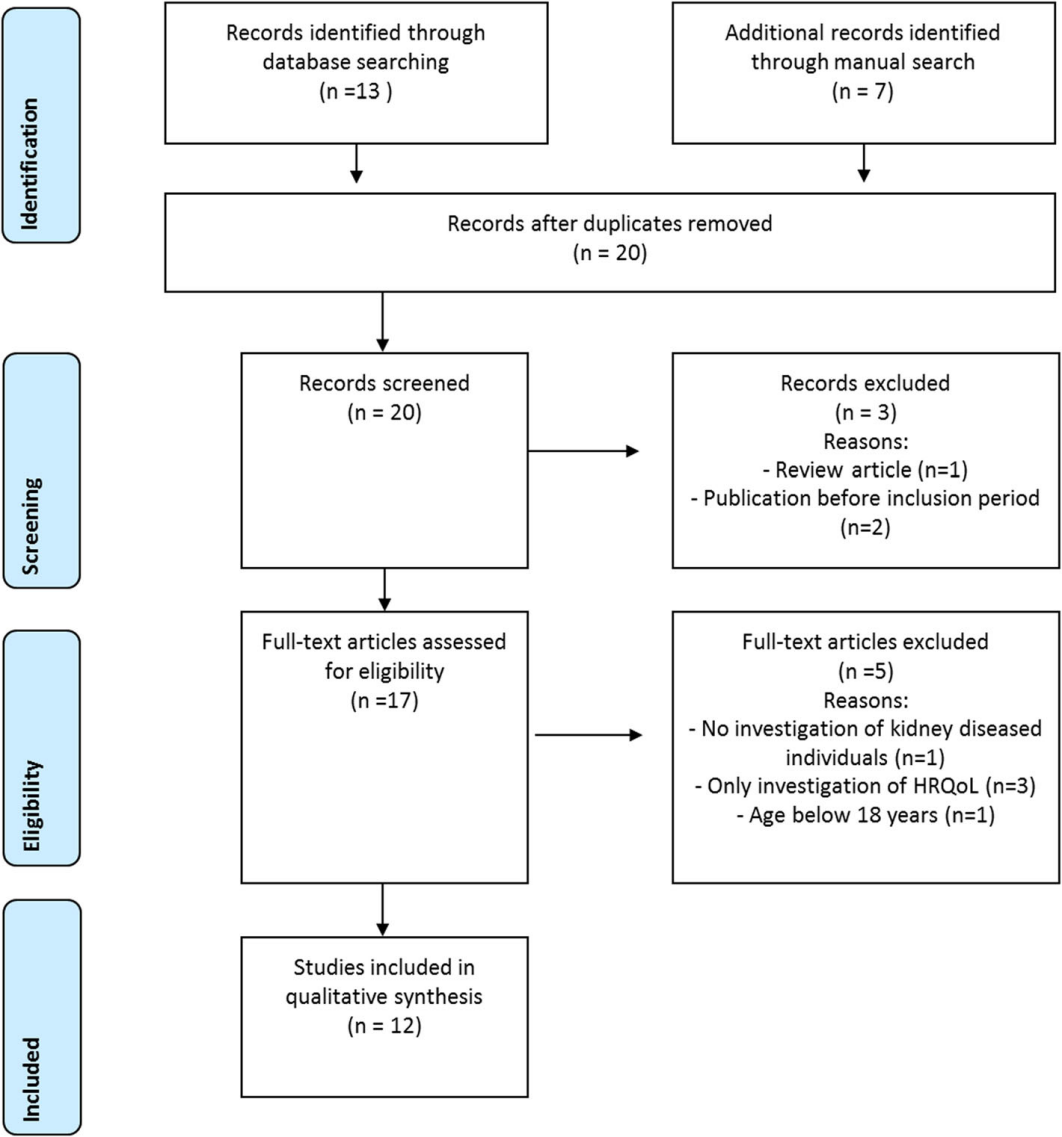
PRISMA diagram for the systematic review process

The search findings according to the PRISMA statement [16] are presented in Fig. 1. A total of 20 studies were identified by systematic search complemented by manual search. Of these findings, three studies were excluded during screening; in one case, the type of article (systematic review) was the reason, and in two cases, the publication time was before the examination period (before 1st January 2009). A total of 17 full-text articles were examined regarding the inclusion and exclusion criteria. Five studies were excluded: one study did not examine end-stage renal diseases, one study included participants younger than 18 years and three studies examined HRQoL but not OHRQoL. Accordingly, a total of twelve clinical investigations were included in the qualitative analysis.

### Characteristics of included studies

Studies from eight different countries were included. The majority (11/12 studies) of all included patients underwent haemodialysis (HD), with a sample size between 47 and 512 participants. In two studies, kidney transplant recipients (KTx) were included; 39 and 51 participants were examined. The study type, mean age, gender and disease duration of the included studies are presented in Table 1. Only one-quarter of the investigations included a healthy control group for comparison of the OHRQoL findings.

**Table 1:** Studies included in this systematic review. Values for age and disease duration are presented as the mean value ± standard deviation, mean value (range) or percentage

Author, year	Disease	Country	No. of patients	Study type	Subjects mean age in years	Disease duration	Female (%)	Control group for OHRQoL
*Guzeldemir* et al. *2009* [17]	HD	Turkey	47	monocentric cross-sectional	46.38 ± 15.10 years	HD: 71.04 ± 1.16 months	48.9%	no
*Hajian-Tilaki* et al. *2014* [18]	HD	Iran	145	monocentric cross-sectional	58.17 ± 17.76 years	HD: 49.33 ± 46.99 months	46.9%	no
*Pakpour* et al. *2015* [19]	HD	Iran	512	multicentre cross-sectional	57.7 ± 17.01	HD: 52.12 ± 29.86 months	37.1%	yes, healthy control *n* = 255, 55.8 ± 15.9 years, 38% female
*Schmalz* et al. *2016* [20]	HD, KTx	Germany	HD: 87, KTx: 39	multicentre cross-sectional	HD: 60.98 ± 14.01 years, KTx: 56.51 ± 11.56 years	n/a	HD: 37.9% KTx: 51.3%	yes, healthy control *n* = 91, age: 58.31 ± 9.91 years, 65.9% female
*Lopez-Pintor* et al. *2017* [21]	HD	Spain	50	monocentric cross-sectional	66.62 ± 13.96 years	HD: 46.02 ± 44.90 months	30%	no
*Lira E Silva* et al. *2017* [28]	HD	Brazil	226	multicentre cross-sectional	55.52 ± 14.70	HD: < 1 year: 17.3%, 1–5 years: 56.6%, > 5 years 26.1%	40.7%	no
*Rodakowska* et al. *2018* [22]	HD	Poland	72	monocentric cross-sectional	63.2 ± 15.2 years	HD: 43.8 (1–264) months	60.7%	no
*Camacho-Alonso* et al. *2018* [23]	HD	Spain	120	multicentre cross-sectional	69.90 ± 11.61 years	HD: < 1 year: 11.7%, 1–2.9 years: 10.8%, 3–4.9 years: 22.5%, 5–9.9 years: 35.8%, > 10 years: 19.2%	31.7%	yes, healthy control *n* = 120, mean age 67.71 ± 8.96, 37.5% female
*Schmalz* et al. *2018* [24]	HD	Germany	210	multicentre cross-sectional	64.92 ± 15.7 years	HD: 0–2 years: 15.3%, 3–5 years: 18.4%, 6–8 years 17.9%, 9–12 years 15.3%, 13–20 years 17.9%, > 20 years: 15.3%	35%	no
*Ruokonen* et al. *2019* [25]	KTx	Finland	51	prospective follow-up cohort study	61 (31–86) years	time since KTx 7.1 (1–11) years	33%	no
*Kahar* et al. *2019* [26]	HD	USA	70	multicentre cross-sectional	≤65 years: 44.1% > 66 years: 55.9%	4.7 ± 7.5 years	39.7%	no
*Oliveira* et al. *2019* [27]	HD	Brazil	180	multicentre cross-sectional	51.98 ± 14.34 years	< 12 months: 23.3%, 12–36 months 32.7%, > 36 months	45%	no

*OHRQoL* oral health-related quality of life, *n/a* not applicable, *HD* haemodialysis, *KTx* kidney transplantation

### Oral health record and findings

As shown in Table 2, the applied oral examinations were heterogeneous across included studies. Each study considered at least one parameter regarding missing and/or remaining teeth or denture wearing. The average number of missing teeth per study varied between 6.53 and 11.71, while the average number of remaining teeth per study varied between 16.9 and 21.7, where applicable. Dental health was frequently assessed, whereby the decayed-, missing- and filled-teeth index (DMF-T) and its components, as well as dental treatment need, were examined. Five studies also evaluated oral hygiene indices, mainly the plaque index (PI) and gingival index (GI). Eight studies examined periodontal parameters, especially the periodontitis severity, community periodontal index (CPI) and/or periodontal probing depth (PPD). The periodontitis prevalence of studies presenting this aspect ranged between 36.3 and 96.6%. As a specific oral health parameter, approximately half of studies reported salivary findings or dry mouth/xerostomia. Table 2 provides a detailed overview of examined oral health parameters and results, if applicable.

**Table 2:** Examined oral health parameters and the main results of oral conditions if they were presented as the mean values ± standard deviation, mean (range) or percentage in the included studies

Author, year	Tooth loss, remaining teeth, dentures	Dental diseases, caries, dental treatment need	Oral hygiene indices	Periodontal parameters, periodontal treatment need	Further oral health parameters
*Guzeldemir* et al. *200 9*[17]	M-T: 6.53 ± 7.16	n/a	PI: 2.21 ± 0.66, GI: 1.24 ± 0.77	PPD: 2.21 ± 0.66, BOP: 33.51 ± 24.58	n/a
*Hajian-Tilaki* et al. *2014* [18]	M-T: 10.06 ± 7.30/6.56 ± 6.51 depending on DM, edentulous: 34.5%, dentures: 44.8%	DMF-T: 15.47 ± 7.85	PI: 2.03 ± 0.95, poor oral hygiene: 66.3%	PDI: 4.09 ± 1.32	Dry mouth: 51.7%, taste change: 35.2%, halitosis: 40%
*Pakpour* et al. *2015* [19]	M-T: 11.71 ± 7.68	D-T: 0.91 ± 1.93, F-T: 7.37 ± 8.02, DMF-T: 20.06 ± 11.16	GI: 1.59 ± 0.97, PI: 1.92 ± 1.28	CPI: 2.34 ± 1.12	n/a
*Schmalz* et al. *2016* [20]	HD: M-T: 9.28 ± 6.49 KTx: M-T: 7.15 ± 6.21	HD: DMF-T: 20.43 ± 5.85, D-T: 2.29 ± 4.13, F-T: 8.86 ± 5.30 KTx: DMF-T: 17.41 ± 5.51, D-T 0.74 ± 0.43, F-T: 9.51 ± 4.23	n/a	HD: Periodontitis: 96.6% KTx: Periodontitis: 87.2%	n/a
*Lopez-Pintor* et al. *2017* [21]	denture wearing: 34%	n/a	n/a	n/a	UWS: 0.16 ± 0.17, SWS: 1.12 ± 0.64, xerostomia VAS: 38.30 ± 15.07
*Lira E Silva* et al. *2017* [28]	dentures: 39.8%	DMF-T: 22.68 ± 8.37	n/a	n/a	No dental visit for 3 years: 44.7%, toothache in previous 6 months: 18.1%, mucosa alterations: 45.6%
*Rodakowska* et al. *2018* [22]	18% edentulous, number of teeth < 20: 76.4%	dental treatment need: 51.4%	PI > 1: 76.3%, GI > 1: 54.2%, OHI-S > 1.2: 60.9%	n/a	Chewing ability: 30.6%, dry mouth: 58.3%
*Camacho-Alonso* et al. *2018* [23]	number of teeth: 18.25 ± 9.32	n/a	n/a	bleeding index: 33.98 ± 33.28, CPITN: 1.43 ± 1.48, PPD: 1.86 ± 1.12, CAL: 1.62 ± 0.85, number of pockets ≥4 mm: 0.58 ± 2.02, number of pockets ≥6 mm: 0.50 ± 1.73, moderate-severe periodontitis: 36.3%	UWS: 6.11 ± 6.11 ml/15 min
*Schmalz* et al. *2018* [24]	remaining teeth: 16.90 ± 8.8	DMF-T: 20.45 ± 6.8, dental treatment need: 56%	n/a	PPD: 3.86 ± 0.95, periodontal treatment need: 88.8%	n/a
*Ruokonen* et al. *2019* [25]	number of teeth: 21.7 ± 6.8	n/a	n/a	n/a	xerostomia 40%, UWS 0.32 ml/min, SWS: 0.95 ml/min
*Kahar* et al. *2019* [26]	edentulous: 17.1%, M-T: 11.3 ± 10.7, number of teeth < 20: 39.1%, number of teeth: 19.7 ± 11.04	D-T: 1.5 ± 2.9, F-T: 3.0 ± 4.5	n/a	CPI: 1.9 ± 1.0	saliva pH: 6.9 ± 1.0
*Oliveira* et al. *2019* [27]	number of teeth < 20: 40.6%	untreated caries: 17.2%	PI > 15%: 74.4%, GBI > 15%: 33.9%	periodontitis: 86.7% moderate-severe	dental care no visit: 30.5%, xerostomia: 35%

*HD* haemodialysis, *KTx* kidney transplantation, *M-T* missing teeth, *D-T* decayed teeth, *F-T* filled teeth, *DMF-T* decayed-, missing- and filled teeth index, *PI* plaque index, *GBI* gingiva bleeding index, *GI* gingival index, *CPI* community periodontal index, *PPD* periodontal probing depth, *UWS* unstimulated whole saliva, *SWS* stimulated whole saliva, n/a: not applicable, *OHI* oral health index

### OHRQoL measurements and results

**Fig. 2:**
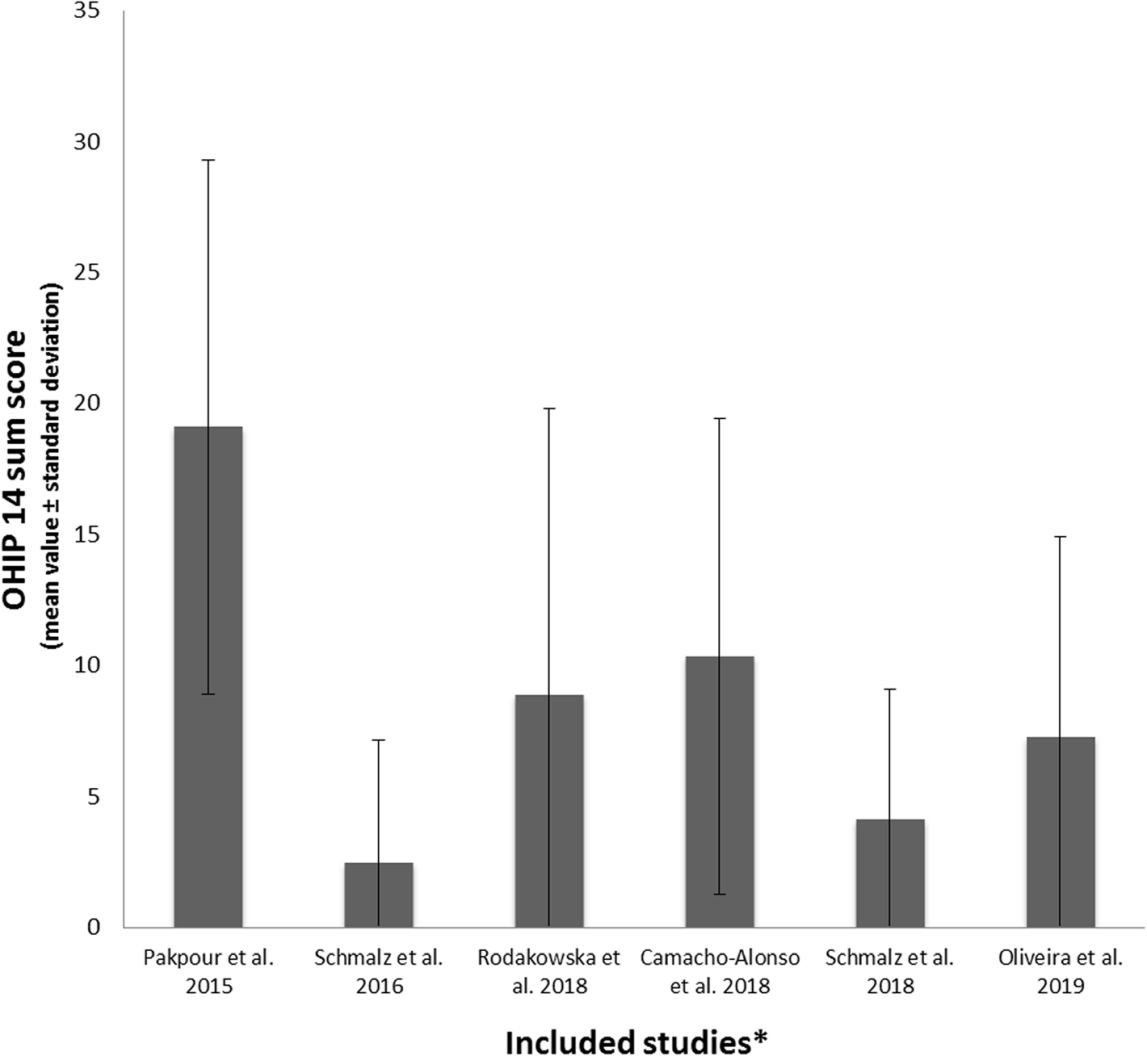
Findings of the included studies. *only studies presenting the OHIP 14 mean value ± standard deviation or median and range, with a 0–4 point scale (0 (“never”) to 4 (“always”)), are displayed in this figure

**Fig. 3:**
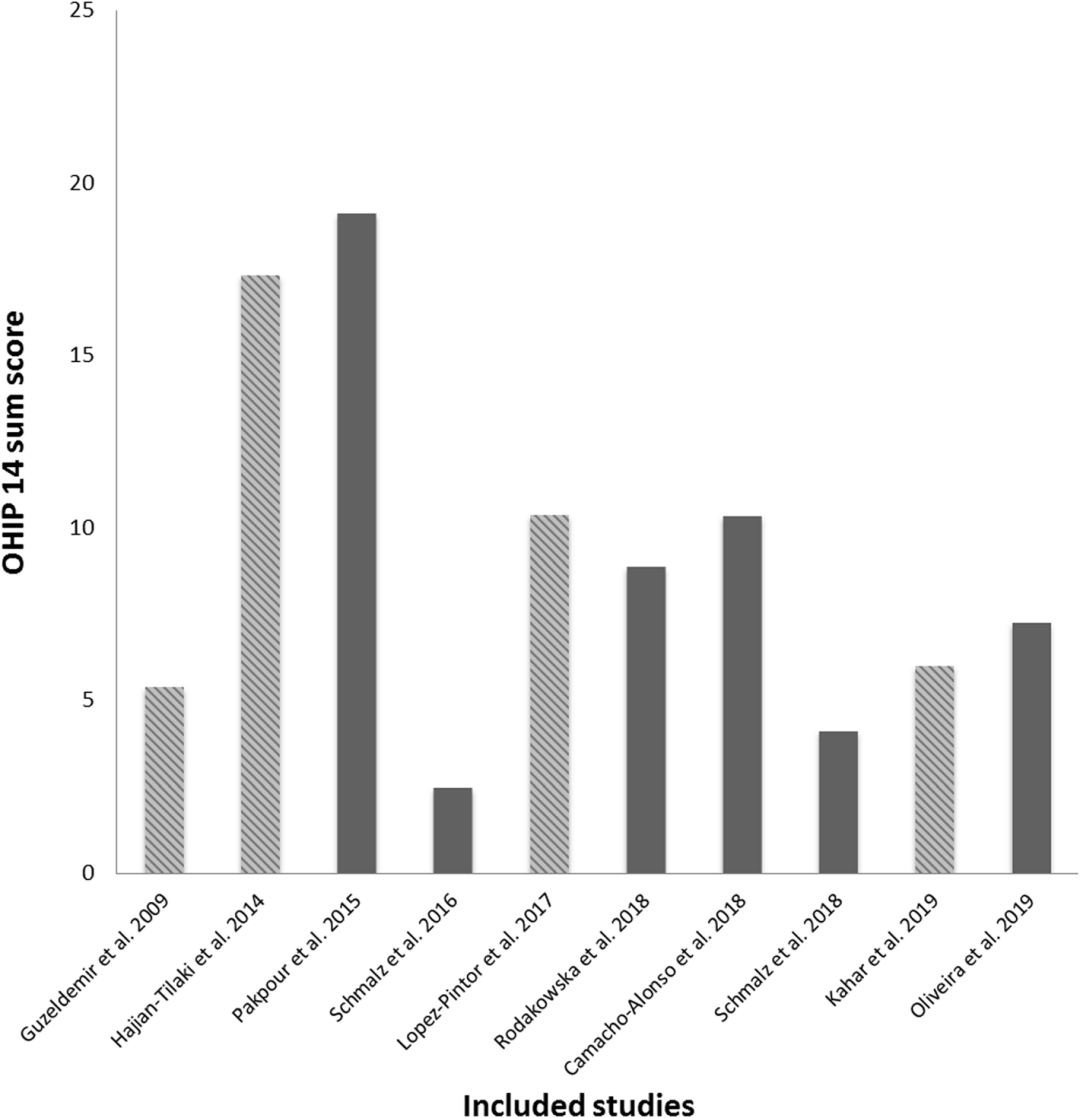
Included studies presenting OHIP 14 values. The diagonally crosshatched bars represent modified OHIP mean values with regard to the rating (0–4 vs. 1–5) and/or the inverse interpretation (0 (“never”) vs. 5 (“never”)). For OHIP 14 findings of the three studies using a scale of 1–5, 14 points were subtracted. Furthermore, the inverse rating, which was 5 (“never”) to 1 (“very often”) was converted by subtracting 14 points first (50/56 instead of 64/70). Afterwards, the difference between the maximum (56) and detected score (50) was calculated (OHIP 14 of 6)

The vast majority of studies (11/12) used the short form of the oral health impact profile (OHIP 14) to assess OHRQoL. Additionally, three studies applied the General Oral Health Assessment Index (GOHAI). One study examined a self-composed oral health quality score questionnaire. Of the three studies comparing OHRQoL with a healthy control, two were able to confirm a worse OHRQoL in HD patients. Regarding the rating of OHRQoL measures, differences in the scores were detected. Three studies rated OHRQoL on a 1–5 point scale instead of 0–4 point scale. Moreover, one study used an inverse interpretation, with higher score representing better OHRQoL. Figure 2 shows the OHIP 14 values of all studies that were presented as either the mean value or median and applied on a rating scale between 0 (“never”) and 4 (“always”). In Fig. 3, the OHIP 14 values of four studies were modified. For the OHIP 14 findings of the three studies using a scale of 1–5, 14 points were subtracted, because even if all questions were answered with “never”, a score of 14 points would have been achieved. Furthermore, the inverse rating, which was 5 (“never”) to 1 (“very often”), was converted by subtracting 14 points first (50/56 instead of 64/70). Afterwards, the difference between the maximum score (56) and detected score (50) was calculated (OHIP 14 of 6). Therefore, Fig. 3 shows the OHIP 14 findings of the included studies, which were adapted as well as possible.

The internal reliability (Cronbach’s α) varied between 0.73 and 0.96 for OHIP 14 and 0.64 and 0.91 for GOHAI. Only three studies examined the relationship between OHRQoL and general HRQoL, whereby two studies were able to confirm this relationship. The majority of studies (10/12) found associations/correlations between OHRQoL and different oral health parameters. The most frequently detected parameters in this context were periodontal and/or oral hygiene parameters. Furthermore, several relationships between OHRQoL and disease related parameters, including diabetes, age, dialysis duration, body-mass-index (BMI) and laboratory values were found. The results of the OHRQoL of the included studies are presented in Table 3.

**Table 3:** Applied assessments for OHRQoL and relevant results for the included studies

Author, year	Assessment of OHRQoL	Internal reliability of OHRQoL measurement Cronbach’s α	OHRQoL worse than control	Association/correlation between OHRQoL and general HRQoL	Association/correlation between OHRQoL and oral health	Association and/or correlation between OHRQoL and disease related parameters
*Guzeldemir* et al. *2009* [17]	OHIP 14: 19.40 ± 7.74 (8–29)* GOHAI: 15.72 ± 8.68 (3–31)	OHIP 14: 0.73, GOHAI: 0.82	n/a	n/a	OHIP 14 and GOHAI: M-T, OHIP 14: PI	OHIP 14: creatinine
*Hajian-Tilaki* et al. *2014* [18]	OHIP 14: 31.32 ± 12.53 (14–68)* GOHAI: 29.07 ± 8.5 (14–50)	OHIP 14: 0.96, GOHAI: 0.85	n/a	n/a	OHIP 14: OHI-S, dry mouth, taste change, halitosis, PI, PDI, DMF-T GOHAI: OHI-S, dry mouth, taste change, PI, PDI, DMF-T	OHIP 14: diabetes
*Pakpour* et al. *2015* [19]	OHIP 14: 19.10 ± 10.21	n/a	yes	PCS and MCS of SF-36 with OHIP 14	DMF-T, PI, CPI, GI, oral behaviour (regular tooth brushing)	age, BMI, diabetes, Kt/v
*Schmalz* et al. *2016* [20]	OHIP 14: HD: 2.46 ± 4.68, KTx: 2.54 ± 3.68	n/a	no	n/a	no	HD: diabetes
*Lopez-Pintor* et al. *2017* [21]	OHIP 14: 24.38 ± 11.98*	n/a	n/a	n/a	xerostomia VAS, xerostomia	no
*Lira E Silva* et al. *2017* [28]	OHIP 14: no total mean value provided. Low impact: 40.7%, medium impact: 32.7%, high impact: 26.5%	n/a	n/a	n/a	history of toothache	age
*Rodakowska* et al. *2018* [22]	OHIP 14: 8.87 ± 10.95 (4; 1–13) GOHAI: 14.71 ± 7.21 (13; 9–19)	OHIP 14: 0.918, GOHAI: 0.637	n/a	n/a	OHIP 14: dental treatment need, chewing ability GOHAI: chewing ability	no
*Camacho-Alonso* et al. *2018* [23]	OHIP 14: 10.34 ± 9.09	n/a	yes	no (HADS)	no	no
*Schmalz* et al. *2018* [24]	OHIP 14: 4.1 (0, 0–5)	n/a	n/a	n/a	PPD	dialysis duration
*Ruokonen* et al. *2019* [25]	oral health quality score (OHQS) 75.1% maximum score	n/a	n/a	15D questionnaire	PPD, PIBI, TDI	n/a
*Kahar* et al. *2019* [26]	OHIP 14: 64 (54.8–68), GOHAI-12: 52 (39.8–56.3)**	OHIP 14: 0.92, GOHAI: 0.91	n/a	n/a	OHIP: remaining teeth, dentate vs. edentulous GOHAI: remaining teeth	GOHAI: age, gender, dialysis duration
*Oliveira* et al. *2019* [27]	OHIP 14: 7.25 ± 7.69	n/a	n/a	n/a	PI, periodontitis severity	no

*n/a* not applicable, *OHIP* oral health impact profile, *GOHAI* general oral health assessment index, *PCS* physical compound summary, *MCS* mental compound summary, *SF-36* short form 36 questionnaire, *HADS* Hospital Anxiety and Depression Scale, *HD* haemodialysis, *KTx* kidney transplantation, *M-T* missing teeth, *DMF-T* decayed-, missing- and filled teeth index, *PI* plaque index, *GBI* gingiva bleeding index, *GI* gingival index, *CPI* community periodontal index, *PPD* periodontal probing depth, *UWS* unstimulated whole saliva, *SWS* stimulated whole saliva, *OHI* oral health index, *BMI* body mass index, *PIBI* periodontal inflammatory burden index, *TDI* total dental index, *OHIP 14 scale of 1-5 instead of 0-4 points, **OHIP 14 scale of 1-5 instead of 0-4 and inverse rating

### OHRQoL subscales

Different subscales of OHRQoL measurements, including OHIP 14 and GOHAI, were presented in the included studies. However, only half of the investigations presented sum scores for the subscales of applied questionnaires. Table 4 shows the findings of these studies, which presented the mean value, median, or percentage of the subscales. Although comparability is limited, psychological/psychosocial impairment and pain were the predominantly affected subscales. The impairment of functional limitations was also present but was more heterogeneous and comparably lower than other subscales.

**Table 4:** Subscales of OHRQoL in included studies. Because different questionnaires were used and several different options of subscales/dimensions exist, the available results are presented if available. Results are given as the mean value ± standard deviation or otherwise as percentage

OHIP 14							
Disease	Functional limitation	Physical pain	Psycho-social discomfort	Physical disability	Psycho-logical disability	Social disability	Handicap
*Lopez-Pintor* et al. 2017* [21]	4.20 ± 2.45	4.04 ± 2.13	3.98 ± 2.54	3.34 ± 2.05	3.26 ± 1.94	2.76 ± 1.68	2.80 ± 1.84
*Lira E Silva* et al. *2017* [28]	1.18 ± 1.07	1.73 ± 1.15	1.11 ± 1.18	1.51 ± 1.10	0.89 ± 1.15	0.23 ± 0.50	0.63 ± 0.81
*Camacho-Alonso* et al. *2018* [23]	1.86 ± 1.81	2.17 ± 2.31	2.08 ± 1.89	1.94 ± 2.14	0.88 ± 1.45	0.74 ± 1.35	0.68 ± 1.38
*Oliveira* et al. *2019* [27] *No/mild or moderate/severe periodontitis*	0.29 ± 1.00/0.48 ± 1.00/0.72 ± 1.16	1.5 ± 2.09/1.54 ± 1.87/2.16 ± 2.44	1.21 ± 1.64/1.51 ± 2.10/2.04 ± 2.44	0.54 ± 1.25/0.56 ± 1.16/0.91 ± 1.78	0.63 ± 1.10/0.94 ± 1.51/1.35 ± 1.90	0.42 ± 0.97/0.55 ± 1.38/0.54 ± 1.32	0.17 ± 0.82/0.62 ± 1.52/0.78 ± 1.68
	**Functional limitation**		**Pain and discomfort**		**Psychological impact**		**Behavioural impact**
*Rodakowska* et al. *2018* [22]	44.4%		52.8%		62.5%		45.8%
	**Oral function**		**Psychosocial impact**		**Pain**		
*Schmalz* et al. *2018* [24]	1.66 (0,0–3)		2.10 (0, 0–3)		0.43 (0, 0–1)		
**GOHAI**							
	**Functional limitation**		**Pain and discomfort**		**Psychological impact**		**Behavioural impact**
*Rodakowska* et al. *2018* [22]	88.9%		84.7%		94.4%		30.6%

*OHIP* oral health impact profile, *GOHAI* general oral health assessment index, *OHIP 14 scale 1-5 instead of 0-4 points

## Discussion

The definition of the FDI World Dental Federation interprets oral health as the synthesis of physical, functional and psychosocial parameters [29]. This current systematic review focused on the OHRQoL of patients with end-stage renal disease undergoing RRT (HD and KTx) to assess these parameters. Therefore, OHRQoL reflects the subjectively perceived impact of oral health on different dimensions of quality of life [7, 9, 30]. Different measurements for the assessment of OHRQoL are available and are normally based on questionnaires asking for functional and psychosocial impacts that patients perceive with respect to their teeth, mouth or dentures [30]. The assessment of OHRQoL allows a shift from traditional physical dental criteria to individual social, emotional and physical functioning of a patient [30]. The OHIP 14, which was mainly used in the included studies, assesses 14 questions about different potential impacts related to oral health on a scale between 0 (“never”) and 4 (“always”). Therefore, higher OHIP 14 scores represent worse OHRQoL (Supplementary Table 1).

Although OHRQoL was the main focus of this review, the physical oral health findings were considered and must be interpreted in the context of their potential clinical relevance for patients. While it is known that patients with RRT suffer from insufficient oral health [10, 31], the impact of oral disease on the mortality of patients is controversial [32–34]. Therefore, different systemic consequences of oral health can be recognized. For instance, periodontal disease can increase the risk of infections, such as pneumonia, in patients with RRT [35]. Especially in the elderly population, tooth loss and being edentulous can be related to malnutrition and mortality [36, 37]. This might be of importance for patients under RRT, because they are predisposed for malnutrition with an increased risk of mortality [38]. Accordingly, increased attention to oral care and sufficient interdisciplinary dental care concepts appear necessary [10, 15].

In general, the included studies showed heterogeneous oral health findings for different parameters. The studies that examined periodontal parameters showed a remarkable periodontal burden or periodontal treatment need [17–20, 23–27]. This finding is relevant because periodontal inflammation can be related to an increased risk of bacteraemia and thus systemic infections [39] or pneumonia caused by aspiration of periodontal bacteria [35]. Therefore, a sufficient, inflammation-free periodontal situation of patients under RRT should be a clinical goal. Only five studies confirmed an impact of periodontal parameters on the OHRQoL of patients under RRT [18, 19, 24, 25, 27]. These results suggest that some patients did not perceive their insufficient periodontal situation as a problem.

Similarly, tooth loss and denture wearing were frequently detected oral health issues. The clinical relevance of these parameters is somewhat limited, because they provide no information regarding what teeth are missing and whether the chewing function is affected. Accordingly, the potential impact on malnutrition would just be speculative in this case. Only the minority of studies found missing/remaining teeth as an influential factor on OHRQoL [17, 26]. This is surprising because tooth loss can be seen as an important parameter related to reduced OHRQoL in systemically healthy individuals [40]. Xerostomia is prevalent in patients under RRT [41] and is also an influential factor on OHRQoL [42, 43]. This parameter has also been rarely assessed, and it was only found to be associated with OHRQoL in two studies [18, 21]. The significance of these findings appears limited, because the examined parameters were quite heterogeneous between studies (see Table 2).

The OHRQoL of the included studies was measured by three different methods. The majority applied OHIP 14, which can be seen as a valid questionnaire to assess OHRQoL [44]. This questionnaire is composed of 14 questions regarding possible impacts that patients perceived as related to their teeth and dentures; these questions were originally scaled between 0 (“never”) and 4 (“very often”) [44]. The second measurement was the GOHAI questionnaire, which was originally composed for geriatric patients [45]. This measurement includes 12 questions with a Likert scale between 1 (“always”) and 5 (“never”) [45]. The third questionnaire was a self-composed oral health quality score, which has been applied to patients after KTx [25]. Four studies applied the OHIP 14 scores but changed the original scoring [17, 18, 21, 26]. All other studies that applied OHIP 14 referred to the original scale between 0 and 4 points [19–24, 27, 28].

To enable comparison to the other studies, we tried to adapt these values as described in the results section. Figure 2 and Table 3 show the originally presented results, while the modified findings are displayed in Fig. 3. One can see that after adaptation of the values, the majority of the results were between 2 and 15 points, while only two Iranian studies having a sum score of approximately 20 (Fig. 3).

However, the adaptation must be seen as a limitation. There seems to be only a slight to moderate reduction in OHRQoL in the included studies; however, the interpretation of the OHRQoL impairment is difficult. Only three studies examined a healthy control group, of which two studies showed worse OHRQoL in HD patients [19, 23]. International reference values for interpretation are not yet available. The studies were performed in different countries, and cultural differences as well as a large influence of age, gender and presence of teeth/prosthodontic treatment on OHRQoL outcome are conceivable [30, 46]. For instance, the general German dental patient population should exhibit a sum OHIP 14 score of 0–4 points depending on full or partial dentition [46]. Only one study [20] found an OHIP 14 within these reference values, while the OHIP 14 scores in the other studies were higher and might be interpreted as an impaired OHRQoL. A reduction in OHRQoL can be the result of different causes, whereby oral health and general HRQoL are major influential factors [47]. As mentioned above, the relationship between OHRQoL and oral health in patients under RRT is heterogeneous and limited by the variety of examined parameters. Accordingly, general HRQoL and disease-related factors must be recognized.

A relationship between general HRQoL and OHRQoL in patients under RRT has been evaluated in only three studies, whereby three different measurements were applied [19, 23, 25]. Two studies confirmed an association between general HRQoL and OHRQoL [19, 25]. This limits the ability to draw meaningful conclusions. Considering OHRQoL to be a part of HRQoL [7, 8, 47], an interrelationship between these parameters seems plausible in patients under RRT. It is known that general HRQoL is reduced in patients under RRT, especially those under HD [4–6]. Therefore, impairment of HRQoL, and in consequence OHRQoL, is conceivable, although it cannot be strongly confirmed by available studies. Furthermore, KTx can improve HRQoL, while oral health problems still exist after KTx [48–50]. The influence of KTx on OHRQoL remains questionable, because there are too few data available. In the included studies, one investigation found comparable OHRQoL between HD and KTx, and another showed a slight improvement of OHRQoL after KTx compared to the predialysis stage [20, 25]. Moreover, the form of dialysis (HD or peritoneal dialysis) might have an impact on both general HRQoL and oral health situation [51, 52]. Data regarding the OHRQoL in this context are not yet available.

The influence of parameters of RRT as well as patient-specific factors on OHRQoL is a further field of interest. Two studies found dialysis duration, i.e., the overall time of patients being under HD, to be related to OHRQoL. This is plausible, as both oral health and HRQoL become worse with prolonged dialysis duration [53, 54]. The association between diabetes mellitus and worse OHRQoL in patients with RRT also seems plausible, because diabetes and related oral alterations are correlated with worse OHRQoL [55]. Furthermore, diabetes could have a negative influence on periodontal health, tooth loss or salivary flow of patients under RRT, although this is discussed controversially in the literature [56, 57]. Although only one large-scale study found a relationship between OHRQoL and BMI, this may further suggest an association between oral inflammation and malnutrition of patients under RRT [58]. This finding may be of high practical relevance, because malnutrition is related to mortality in dialysis patients [38]. Altogether, data regarding the influence of disease-related parameters in patients with RRT with OHRQoL are rare and heterogeneous. Nevertheless, these factors are of potential relevance in the complex evaluation of OHRQoL and for the clinical dental care of these patients.

**Fig. 4:**
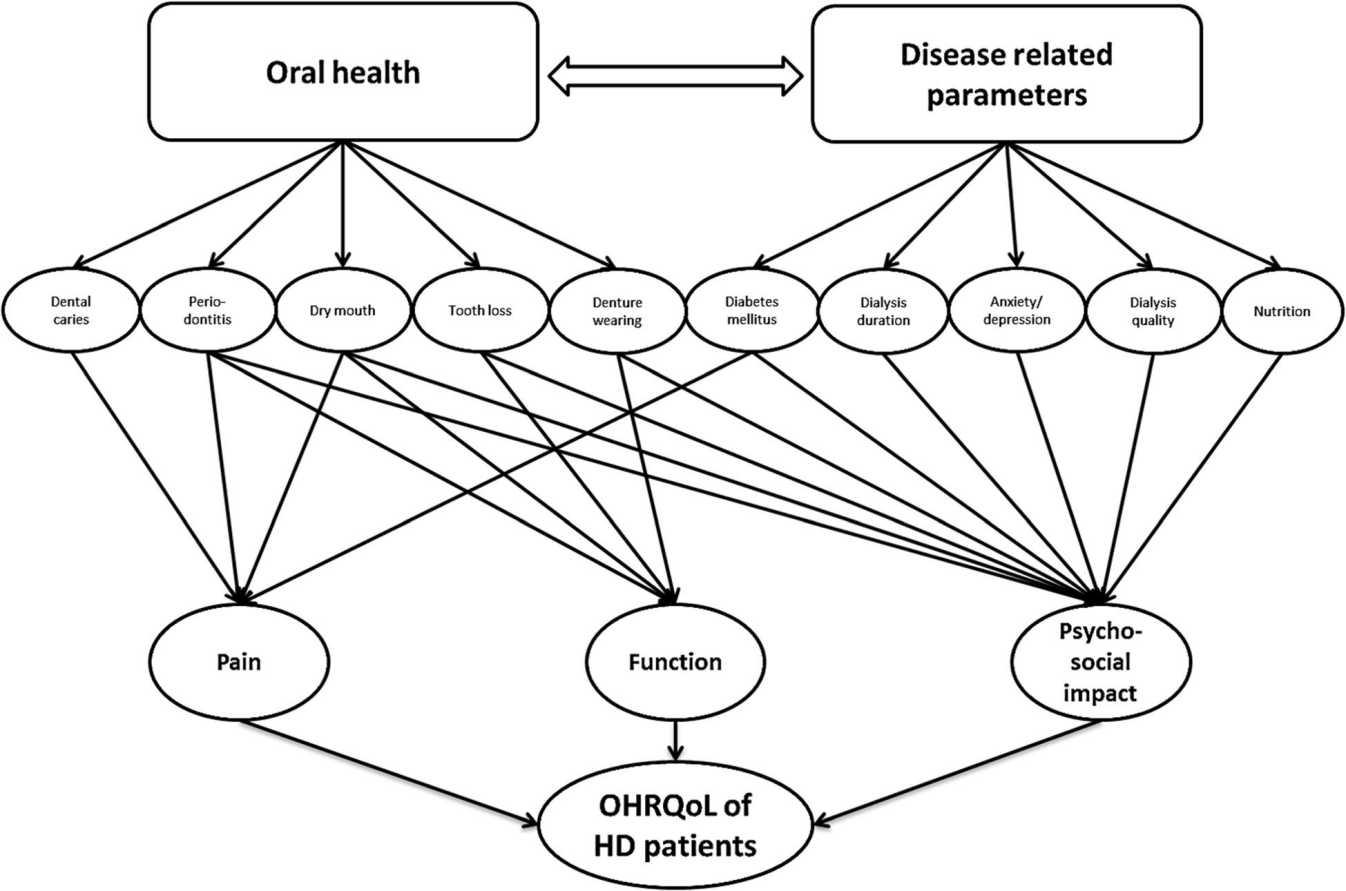
OHRQoL of patients undergoing HD is complex. Different oral health as well as disease-related parameters affect different dimensions of OHRQoL. Furthermore, disease-related factors affect the oral health situation of the patients and, in consequence, OHRQoL. While pain and function is primarily affected by oral health, the psychosocial impact is affected by oral conditions and disease related parameters. Diabetes as a disease-related parameter can cause diabetic neuropathy in the oral cavity and might influence the pattern pain. This figure is just a simplified construct; the clinical interrelation is far more complex

To obtain a deeper understanding of the OHRQoL of patients under RRT, different subscales of OHRQoL might give information regarding the impairment of functional/physical and/or psychosocial/psychological patterns. The interpretability of these findings is limited, because only half of the studies reported subscales, and no comparison to healthy controls was performed (see Table 4). Accordingly, the results must be interpreted carefully. In general, an impairment of all three major dimensions, including oral function, psychosocial impact and pain, seems present. Therefore, psychosocial impairment appears to be the most pronounced dimension. It is known that psychosocial factors are predictors of HRQoL in dialysis patients [54]. The psychological burden caused by dialysis therapy as well as the occurrence of fatigue, anxiety and depression in patients under RRT has been reported [54, 59]. Furthermore, periodontitis severity and tooth loss can be related to emotional well-being and psychosocial impacts [60, 61]. Accordingly, the effect of psychosocial impact might be explained by a combination of RRT and oral health related issues. This assumption is supported by the strongest association between psychosocial impacts with dialysis duration and periodontitis severity, as presented in two included studies [24, 27]. The OHRQoL of patients under RRT appears to be complex and potentially influenced by different disease and oral health-related parameters (Fig. 4); however, the majority of potential conclusions remains speculative.

Based on the results of included studies, several implications for the clinical dental care of patients under RRT can be provided. The improvement of the HRQoL of dialysis patients is an important challenge [62]. Oral health parameters – especially tooth loss, periodontitis severity and reduced salivary flow – should be recognized by dental and medical staff. To decrease the risk of systemic infections/complications and malnutrition, an improvement to the oral health of patients under RRT seems mandatory. Therefore, a multidisciplinary care concept is needed. Therefore, in addition to pain and physical issues, the consideration of psychosocial factors is recommended. The dental care of patients with RRT should not only be a physical, surgical treatment but also part of comprehensive medical care. An appropriate sensibilization for the relevance of oral care is needed, because patients have a high burden due to their general disease/therapy and rate oral health as a field of low importance [63]. Moreover, patients’ emotional situation and well-being should be recognized in dental care within an interdisciplinary model.

Furthermore, several recommendations for future research of the OHRQoL of patients under RRT can be provided. The reliability of the applied questionnaires, especially OHIP 14, was appropriate (Cronbach’s α 0.73–0.96). This questionnaire can be recommended, but the original scoring [44] should be applied to ensure comparability with the literature. Moreover, clinical oral examination should include remaining teeth, periodontal diseases and salivary flow, which should be investigated in relation to OHRQoL. The assessment of HRQoL and disease related parameters is also recommended. The analysis of OHRQoL should be performed for sum score and subscales. Reference values for patients under RRT would also be helpful to interpret the findings. Additionally, a minimally important difference to interpret the effects on OHRQoL [64] should be determined in future.

### Strengths and limitations

This systematic review followed the PRISMA statement and was performed by two independent reviewers. Furthermore, it is the first review focusing on the OHRQoL of patients under RRT. Several limitations need to be addressed. The heterogeneity of included studies must be mentioned. Different countries with different health-care systems must be mentioned. Additionally, different reporting of OHRQoL limits the comparability. Although we attempted to adapt the results for comparison in this review, this study has only limited meaningfulness. The analysis was only qualitative, and no meta-analysis was performed. Moreover, the body of literature is quite small. Thus, only 12 studies were included in this systematic review. The inclusion of both HD and KTx and the absence of findings for patients with peritoneal dialysis limits the ability to draw conclusions on the OHRQoL of patients under RRT. Because the majority of studies examined patients under HD, the conclusions made are primarily supported for this type of RRT, while for the other types of RRT, more studies would be needed. Furthermore, longitudinal data regarding changes to OHRQoL during RRT and potential positive effects of dental care measures are not yet available. Only one study followed a prospective design from predialysis to kidney transplantation [25].

## Conclusions

Patients under RRT (HD and KTx) suffer from a reduced OHRQoL, which is potentially influenced by oral health and disease-related parameters in a complex interrelationship. Improvement in dental care is needed, whereby an interdisciplinary model is needed, which should consider both physical and psychosocial issues. Within a multidisciplinary team, including dental, medical and psychological staff, patients should be sensibilized for oral health and receive individualized therapy, if necessary. Future studies in the field should use comparable methodology, should consider oral health alongside disease-related parameters and should aim to determine the reference values and minimally important differences of OHRQoL measurement in patients under RRT.

## Supplementary information

**Additional file 1: Table S1.** Questions of the short form of oral health impact profile (OHIP 14), which has mainly been applied in the included studies to assess OHRQoL [44]. Each question can be answered on a scale between 0 (“never”) to 4 (“always”). Accordingly, higher OHIP 14 scores represent worse OHRQoL.

## Data Availability

All data generated or analysed during this study are included in this published article.

## Abbreviations

CPI: Community periodontal index
D-T: Number of decayed teeth
DMF-T: Decayed-, missing- and filled teeth index
F-T: Number of filled teeth
GI: Gingival index
GOHAI: General oral health assessment index
HD: Haemodialysis
KTx: Kidney transplantation
M-T: Number of missing teeth
OHIP: Oral health impact profile
OHRQoL: Oral health-related quality of life
PI: Plaque index
PPD: Periodontal probing depth
RRT: Renal replacement therapy
