# Supplementary material for: Oral health-related quality of life in adult patients with end-stage kidney diseases undergoing renal replacement therapy – a systematic review
Source: BMC Nephrol. 2020 Apr 29;21:154. doi: 10.1186/s12882-020-01824-7 (PMC7191826; doi:10.1186/s12882-020-01824-7)
Supplement: Supplementary file 1 — Additional file 1: Table S1. Questions of the short form of oral health impact profile (OHIP 14), which has mainly been applied in the included studies to assess OHRQoL [44]. Each question can be answered on a scale between 0 (“never”) to 4 (“always”). Accordingly, higher OHIP 14 scores represent worse OHRQoL. [file 12882_2020_1824_MOESM1_ESM.docx]

**Supplementary Table 1:** Questions of the short form of oral health impact profile (OHIP 14), which has mainly been applied in the included studies to assess OHRQoL [42]. Each question can be answered on a scale between 0 (“never”) to 4 (“always”). Accordingly, higher OHIP 14 scores represent worse OHRQoL.

| **Question** | **Subscales applied in included studies** | |
| --- | --- | --- |
| Have you had trouble pronouncing any words because of problems with your teeth, mouth or dentures? | Functional limitation | Functional limitation |
| Have you felt that your sense of taste has worsened because of problems with your teeth, mouth or dentures? | Functional limitation | Functional limitation |
| Have you had painful aching in your mouth? | Physical pain | Pain and discomfort |
| Have you found it uncomfortable to eat any foods because of problems with your teeth, mouth or dentures? | Physical pain | Pain and discomfort |
| Have you been self-conscious because of your teeth, mouth or dentures? | Psychological discomfort | Psychological impacts |
| Have you felt tense because of problems with your teeth, mouth or dentures? | Psychological discomfort | Psychological impacts |
| Has your diet been unsatisfactory because of problems with your teeth, mouth or dentures? | Physical disability | Behavioural impacts |
| Have you had to interrupt meals because of problems with your teeth, mouth or dentures? | Physical disability | Behavioural impacts |
| Have you found it difficult to relax because of problems with your teeth, mouth or dentures? | Psychological disability | Psychological impacts |
| Have you been slightly embarrassed because of problems with your teeth, mouth or dentures? | Psychological discomfort | Psychological impacts |
| Have you been slightly irritable with other people because of problems with your teeth, mouth or dentures? | Social disability | Behavioural impacts |
| Have you had difficulty doing usual jobs because of problems with your teeth, mouth or dentures? | Social disability | Behavioural impacts |
| Have you felt that life in general was less satisfying because of problems with your teeth, mouth or dentures? | Handicap | Psychological impacts |
| Have you been totally unable to function because of problems with your teeth, mouth or dentures? | Physical disability | Behavioural impacts |
